# Intersecting social determinants of health, multimorbidity and quality of life in people of Black ethnicities with HIV in South London

**DOI:** 10.1097/QAD.0000000000004343

**Published:** 2025-09-17

**Authors:** Luxsena Sukumaran, Lourdes Dominguez-Dominguez, Lisa Hamzah, Jia Liu, Heidi Lempp, Elena Nikiphorou, Caroline A. Sabin, Frank A. Post, Shema Tariq

**Affiliations:** a Institute for Global Health, University College London; b National Institute for Health and Care Research (NIHR) Health Protection Research Unit (HPRU) in Blood-borne and Sexually Transmitted Infections at University College London; c Berkshire Healthcare NHS Foundation Trust, Slough; d St Georges University Hospital NHS Foundation Trust; e GKT School of Medical Education, Faculty of Life Sciences and Medicine; f Centre for Rheumatic Diseases, School of Immunology and Microbial Sciences, Faculty of Life Sciences and Medicine; g Centre for Education, King's College London; h Department of Sexual Health and HIV, King's College Hospital NHS Foundation Trust, London, UK.

**Keywords:** Black African, comorbidity, deprivation, HIV, mixed-methods study, multimorbidity, quality of life, social determinants of health

## Abstract

**Background:**

Social determinants of health (SDoH) impact health outcomes and rarely exert their influence in isolation. We examined associations between SDoH patterns, multimorbidity and quality of life (QoL) in people of Black ethnicities with HIV in England.

**Methods:**

This mixed-methods study comprised questionnaires, focus group discussions and semi-structured interviews with staff members from a community-based organization. We used principal component analysis to identify patterns of SDoH and *z* scores to describe the burden of each pattern. Associations between SDoH burden scores, multimorbidity and QoL (EQ-5D) were assessed using logistic regression, adjusting for sex and age.

**Results:**

Amongst 340 participants [median (interquartile range, IQR) age 52 (45–57) years, 54% women, 95% HIV RNA <200 copies/ml], we identified three SDoH patterns: livelihood (food, employment and financial insecurity, loneliness and isolation), shelter/displacement (housing, migration and food insecurity) and social exclusion (discrimination, loneliness and isolation). An increase in SDoH *z* scores was associated with higher odds of multimorbidity [livelihood: adjusted odds ratio (aOR) 2.09 (1.63–2.69), shelter/displacement: 1.41 (1.12–1.78), social exclusion: 1.78 (1.40–2.26)]. Higher livelihood and social exclusion *z* scores correlated with all QoL domains (*P* < 0.001), and shelter/displacement was associated with problems with usual activity [aOR 1.29 (1.04–1.61), *P* = 0.02] and pain/discomfort [1.29 (1.05–1.58), *P* = 0.02]. Qualitative findings supported the quantitative findings whilst providing further context on how SDoH intersect and shape health.

**Conclusion:**

This study highlights how SDoH intersect and are associated with multimorbidity and lower QoL in people of Black ethnicities living with HIV. These findings emphasize the need for comprehensive, biopsychosocial interventions to address health inequities in this population.

## Introduction

In many settings, the widespread use of antiretroviral therapy (ART) has transformed HIV into a long-term, manageable condition, significantly improving the life expectancy of people with HIV [1]. However, with increased longevity, people with HIV are more likely to encounter age-related comorbidities, including cardiovascular disease (CVD), malignancies, renal disease and neurocognitive impairment, which may arise earlier and progress more rapidly compared to those without HIV [2–4]. The co-existence of multiple long-term conditions (termed multimorbidity), presents new clinical challenges in this population, including higher healthcare costs, polypharmacy and reduced quality of life (QoL) [5–8]. The risk of multimorbidity may be compounded by co-existing social marginalization and the impact of social determinants of health (SDoH), which can also affect engagement in care.

The WHO defines SDoH as the conditions in which individuals are born, grow, live, work, and age, shaped by societal systems and policies that influence health outcomes [9]. These determinants exist at individual, interpersonal, clinical and structural levels, and include income, education, housing, employment and social inclusion [10]. Individuals with HIV often face disproportionate, intersecting social disadvantages, such as lower socioeconomic status, structural racism and HIV-related stigma. These factors affect their ability to manage HIV and contribute to disparities in engagement with care, ART adherence and overall well being [11–13].

Marginalized communities, particularly racially and ethnically minoritized groups, often experience compounding social challenges that exacerbate health disparities. Despite representing around 4% of the UK population, one-third of those in HIV care were of Black African or Black Caribbean ethnicity in 2023 [14]. These groups are more likely to be diagnosed late, face barriers to care, disengage from services and experience viral rebound in the UK [15]. These disparities may be driven by factors, including structural racism, immigration-related fears, stigma (both HIV-related and cultural), socioeconomic disadvantages and mistrust of healthcare services due to historical and ongoing discrimination [16,17]. Despite these well documented health inequities, research gaps remain in understanding how SDoH intersect to affect multimorbidity and QoL in these populations. Some studies highlight the influence of social context on QoL among racially marginalized people with HIV. For example, a study among low-income, urban African American individuals with HIV and a history of injection drug use found HIV-related stigma and negative social support to be key latent factors linked to reduced QoL [18]. However, majority of existing studies focus on single determinants, such as food or housing insecurity [19–21], which may not fully capture their complex and intersecting effects on health.

We examined the associations between SDoH patterns, comorbidities, multimorbidity and QoL using quantitative methods, and explored the lived experiences of people of Black ethnicities with HIV in South London, UK, an area with high HIV prevalence and socioeconomic deprivation [22], through qualitative methods.

## Methods

This mixed-methods study comprised quantitative questionnaires (completed by individuals attending HIV care), routine clinical data and qualitative accounts obtained via focus group discussions and semi-structured interviews (SSIs) in a community-based organization (CBO). The quantitative and qualitative studies drew from different participant pools, NHS and CBO networks, respectively. We applied the Priority Sequence Model [23], in which quantitative and qualitative methods are used sequentially and complementarily, focusing primarily on quantitative results and using qualitative data to contextualize our findings.

### Quantitative study participants and procedures

The Genetic Markers of Kidney Disease Progression in People of African Ancestry (GEN-AFRICA) study is a cross-sectional cohort study investigating genetic factors associated with chronic kidney disease (CKD) in the UK [24]. Cohort characteristics and eligibility are detailed elsewhere [24]. Between May 2018 and February 2020, 2468 adults of Black ethnicities (Black African, Black Caribbean or other) with HIV, aged at least 18 years, were recruited from HIV and renal clinics at nine sites across England.

A subset (*n* = 398) was recruited into the nested CKD-AFRICA sub-study, which aimed to identify clinical and sociodemographic risk factors for CVD, diabetes and kidney disease. Inclusion criteria were: living with HIV; aged 30–65 years; and previous participation in the GEN-AFRICA study at one of three South London sites. Participants attended a single study visit between September 2020 and January 2022 and received £25 for time and travel. Participants provided written informed consent. Ethical approval was granted by the National Health Service Research Ethics Committee (20/LO/0946) and the Health Research Authority [Integrated Research Application System (IRAS) 278244].

### Quantitative social determinants of health variables

Self-reported data on employment, education, housing, income, food security, immigration status, caring responsibilities and social support were collected using validated measures wherever possible. For the present analysis, SDoH were defined using composite or simple parameters, including food insecurity, housing insecurity, migration status insecurity, financial insecurity, job insecurity, low education status, social isolation and discrimination (see Supplementary Table 1 for definitions and recall periods) [22]. As UK clinics provide free, HIV care irrespective of migration status, healthcare access was not considered a variable of interest.

### Quantitative outcome variable

Participants self-reported their medical history, including diagnoses of CVD, diabetes, kidney disease, lung disease, pain, mental health and other long-term conditions (definitions in Supplementary Table 2). These comorbidities were selected for their prevalence and clinical significance among people of Black ethnicities and those with HIV [24,25].

Multimorbidity was defined as the presence of at least two of the following: CVD, diabetes, kidney disease, lung disease, poor mental health and chronic pain. Health-related QoL (HRQoL) was measured using the validated EQ-5D-5L questionnaire [26], which assesses five domains (mobility, self-care, usual activities, pain/discomfort and anxiety/depression). HRQoL was analysed as a binary variable, that is, no problems vs. any problems in each domain, due to the relatively small sample size. EQ-5D health state index scores were also calculated using the England specific value set, ranging from below 0 to 1, with higher scores indicating better self-perceived health [27]. These scores were analysed as a continuous outcome to capture overall variation in HRQoL.

### Statistical analysis

Principal component analysis (PCA) was used to identify distinct patterns of SDoH that commonly co-occur within the study population. Pairwise associations between the eight SDoH were examined using Somers’ *D* statistic [28]. PCA was then applied to the matrix containing significant associations to identify principal components, or underlying patterns. *Oblimin* rotation was used to allow individuals to be affected by multiple SDoH patterns. The optimal number of components was determined using a scree plot and the very simple structure (VSS) criterion. SDoH with factor loadings at least 0.25 were considered strongly associated with a pattern. For each pattern, individual-level burden scores were calculated by summing the presence of each SDoH (coded as 1 or 0), weighted by its corresponding PCA factor loading. These scores were then standardized for ease of interpretation, with higher *z* scores (>0) indicating a greater burden relative to the sample mean.

Logistic regression was used to describe associations between SDoH patterns and individual comorbidities, multimorbidity and HRQoL. Associations with EQ-5D index scores were assessed using linear regression. All multivariable models adjusted for age and sex at birth *a priori*, with estimates reported as adjusted odds ratios (aOR) or adjusted beta estimates (aBeta) with 95% confidence intervals (95% CI). Statistical significance was set at *P* < 0.05. The analyses were performed in R V4.2.4 (R Foundation for Statistical Computing, Vienna, Austria).

### Qualitative study design

A secondary analysis of qualitative data (collected in 2022) was conducted following completion of the quantitative component, in line with the Priority Sequence design [23]. Details of the original data collection and analysis are described elsewhere [10]. Briefly, between May and July 2022, we conducted four focus group discussions with individuals living with HIV of Black ethnicities (*n* = 20), and SSIs with staff (*n* = 4) from a collaborating CBO. FG participants were invited through the CBO. Inclusion criteria included age 35–65 years, living with HIV, self-identifying as Black, and living with at least one long-term condition (LTC) in addition to HIV. The characteristics of focus group discussion participants are reported elsewhere [10]. Focus group discussion topic guides, co-developed by the CBO and research team, explored experiences of living with LTCs, HIV's impact on these conditions, and effects on physical and mental health, self-management and use of support services. Focus group discussions and SSIs were conducted in person or online (via a video conferencing platform), with audio transcribed verbatim by a professional agency and checked for accuracy by the research team.

The original analysis, conducted as a standalone qualitative project, adopted a phenomenological approach. Two researchers conducted reflexive, inductive thematic analysis in NVivo 12 [29], generating initial codes, before developing key themes and subthemes. A third external qualitative researcher independently analysed data from two SSIs and one focus group discussion, refining themes further. For the present study, we used the same thematic approach but integrated the analysis with quantitative findings, focusing on how individuals manage HIV alongside other LTCs, and how SDoH patterns intersect to influence health outcomes and QoL. This iterative process allowed us to contextualize our quantitative findings. Findings were presented to community members and staff for respondent validation [30]. Ethics approval was granted by the Kings College London Research Ethics Committee (Reference HR/DP-21/22-26315).

## Results

### Characteristics of quantitative study participants

Of the 398 participants with HIV enrolled in the quantitative study, 340 (85.4%) had complete SDoH data. Characteristics of the final analytic sample were comparable to the overall cohort (Supplementary Table 3). Overall, the median [interquartile range (IQR)] age was 52 (45–57) years, 53.5% were women and 9.2% were current smokers (Table 1). Most participants were on ART (99.7%) and had an undetectable viral load (<200 copies/ml; 95.3%). The median (IQR) CD4^+^ T-cell count and years since HIV diagnosis were 544 (368–749) cells/μl and 14 (9–18) years, respectively.

**Table 1:** Baseline socio-demographic, lifestyle and HIV-related characteristics among all CKD-AFRICA substudy participants.

Characteristic *n* (%) or median (IQR)	Total (*n* = 340)
Age (years)	52 (45–57)
Gender	
Male	158 (46.5)
Female	182 (53.5)
Region of birth	
Sub-Saharan Africa	247 (72.7)
Caribbean	32 (9.4)
UK/other	61 (17.9)
BMI (kg/m^2^)	29.9 (26.6–34.3)
Current smoker	30 (9.2)
HIV-related factors	
Years since HIV diagnosis	14 (9–18)
On ART	339 (99.7)
Recent CD4^+^ T-cell count (cells/μl)	544 (368–749)
Nadir CD4^+^ cell count	151 (62–275)
HIV RNA (<200 copies/ml)	324 (95.3)
Socioeconomic factors	
Financial insecurity	178 (52.4)
Food insecurity	75 (22.1)
Housing insecurity	37 (10.9)
Migration status insecurity	42 (12.4)
Job insecurity	95 (27.9)
Low education status	108 (31.8)
Loneliness and isolation	85 (25.0)
Discrimination and unfair treatment	114 (33.5)
Number of SDoH reported	2 (1–3)
None	59 (17.4)
One or more	281 (82.6)
Multimorbidity^a^	178 (52.4)
Individual comorbidities	
Hypertension	180 (52.9)
CVD	29 (8.5)
Diabetes	63 (18.5)
Kidney disease	91 (26.8)
Lung disease	53 (15.6)
Chronic pain	87 (25.6)
Mental health	106 (31.2)

CVD, cardiovascular disease; IQR, interquartile range; SDoH, social determinants of health.

a Defined as the presence of more than two of the following comorbidities: cardiovascular disease, diabetes, kidney disease, lung disease, poor mental health and chronic pain.

### Associations between social determinants of health

The prevalence of individual SDoH ranged from 10.9% (unstable housing) to 52.4% (financial insecurity). Discrimination (33.5%), low education status (31.8%) and employment insecurity (27.9%) were also common (Table 1). Overall, 82.6% reported at least one SDoH, with a median of 2 (IQR 1–3) per individual.

**Fig. 1:**
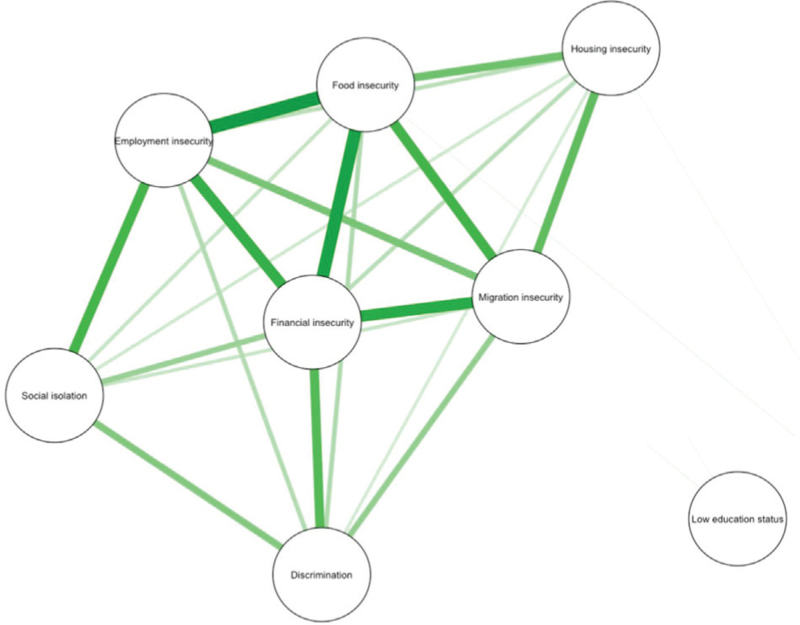
Significant nonrandom associations between social determinants of health, measured using the Somers’ *D* statistic.

Several SDoH exhibited significant associations, including employment, food, financial and migration insecurity (Fig. 1 and Supplementary Table 4). Social isolation was linked to employment insecurity and discrimination, and housing insecurity was associated with food and migration insecurity. In contrast, low education status showed no strong associations with any of the other SDoH and was excluded from the PCA.

The thickness of the line is directly proportional to the absolute value of the Somers’ *D*.

### Patterns of social determinants of health

The PCA identified three SDoH patterns, explaining 60.9% of the total variance (Table 2). The first pattern, which we termed ’livelihood’, accounted for 33.5% of the variance and encompassed employment, food and financial insecurity and social isolation. The second pattern, 'shelter/displacement’, accounted for 14.5% of the variability and included housing, migration, and food insecurity. The third pattern (12.9% of total variability) showed strong correlations with discrimination, loneliness and isolation, and was termed ‘social exclusion’.

**Table 2:** Social determinants of health patterns identified by principal component analysis in CKD-AFRICA participants, explaining 60.9% of the total variability.

Component (% of variance)	Label	Comorbidities (with correlation ≥0.25 with component)
1 (33.5)	Livelihood	Food insecurity (0.66), employment insecurity (0.85), financial insecurity (0.50), loneliness and isolation (0.43)
2 (14.5)	Shelter/displacement	Housing insecurity (0.74), migration insecurity (0.64), food insecurity (0.38)
3 (12.9)	Social exclusion	Discrimination (0.85), loneliness and isolation (0.63)

**Fig. 2:**
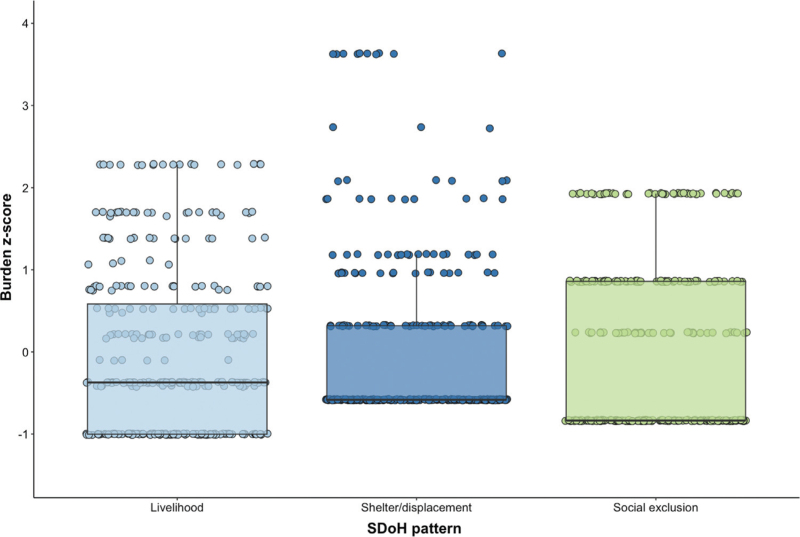
Distribution of social determinants of health patterns’ burden *z* scores in CKD-AFRICA participants.

The highest median (IQR) burden *z* score was observed for the ‘livelihood’ [−0.37 (−1.00 to 0.64)] pattern, followed by the 'shelter/displacement’ [−0.58 (−0.58 to 0.32)] and 'Social exclusion’ [−0.83 (−0.83 to 0.86)] patterns (Fig. 2). The ’livelihood’ scores correlated strongly with 'shelter/displacement’ (*r* *=* 0.50) and 'social exclusion’ (*r* = 0.41) scores (all *P*s < 0.001) (Supplementary Table 5). Similarly, 'shelter/displacement’ scores were moderately correlated with 'social exclusion’ (*r* = 0.24) scores (*P* < 0.001).

### Associations with individual comorbidities and multimorbidity

At baseline, 52% of participants had multimorbidity, with the most common comorbidities being hypertension (53%), mental health disorders (31%) and chronic kidney disease (27%) (Table 1). There were no associations between any of the SDoH patterns, and either hypertension, diabetes, chronic kidney disease or CVD (Table 3). In contrast, higher ‘livelihood’ *z* scores were associated with lung disorders [adjusted odds ratio [aOR] 1.59, (95% CI 1.20–2.12)], chronic pain [aOR 1.83 (1.42–2.36)] and mental health disorders [aOR 2.32 (1.80–2.99)]. Similarly, higher ‘shelter/displacement’ and ‘social exclusion’ *z* scores were associated with mental health disorders [shelter/displacement: aOR 1.32 (1.06–1.66) and social exclusion: aOR 2.36 (1.84–3.03)], with ‘social exclusion’ also associated with chronic pain [aOR 1.24 (0.98–1.58)]. Significant associations were also observed between all three SDoH patterns [‘livelihood’: aOR 2.09 (1.63–2.69), ‘shelter/displacement’: aOR 1.41 (1.12–1.78), and ‘social exclusion’: aOR 1.78 (1.40, 2.26)] and multimorbidity.

**Table 3:** Associations between social determinants of health patterns and individual comorbidities and multimorbidity, assessed using logistic regression models.

	Hypertension		Diabetes		Chronic kidney disease		Cardiovascular disease	
Social determinants of health patterns OR (95% CI) (*P* value)	Unadjusted	Adjusted^a^	Unadjusted	Adjusted^a^	Unadjusted	Adjusted^a^	Unadjusted	Adjusted^a^
Livelihood	0.86 (0.69–1.07), *P* = 0.17	0.87 (0.69–1.10), *P* = 0.24	1.15 (0.88–1.50), *P* = 0.30	1.18 (0.90–1.56), *P* = 0.24	1.13 (0.90–1.44), *P* = 0.30	1.15 (0.90–1.47), *P* = 0.25	1.36 (0.95–1.94), *P* = 0.09	1.40 (0.97–2.02), *P* = 0.08
Shelter/displacement	0.81 (0.65–1.00), *P* = 0.05	0.90 (0.71–1.14), *P* = 0.37	1.14 (0.89–1.48), *P* = 0.30	1.27 (0.97–1.66), *P* = 0.08	1.10 (0.87–1.38), *P* = 0.43	1.17 (0.92–1.48), *P* = 0.20	0.86 (0.56–1.33), *P* = 0.50	0.95 (0.61–1.48), *P* = 0.81
Social exclusion	0.90 (0.72–1.11), *P* = 0.32	0.90 (0.72–1.14), *P* = 0.39	0.84 (0.63–1.12), *P* = 0.23	0.85 (0.63–1.14), *P* = 0.27	1.11 (0.88–1.41), *P* = 0.39	1.13 (0.89–1.44), *P* = 0.33	0.94 (0.64–1.39), *P* = 0.77	0.96 (0.65–1.44), *P* = 0.86

	Lung disorders		Chronic pain		Mental health disorders		Multimorbidity	
Social determinants of health patterns	Unadjusted	Adjusted^a^	Unadjusted	Adjusted^a^	Unadjusted	Adjusted^a^	Unadjusted	Adjusted^a^
Livelihood	1.56 (1.18–2.05), *P* = 0.002	1.59 (1.20–2.12), *P* = 0.001	1.81 (1.42–2.31), *P* < 0.001	1.83 (1.42–2.36), *P* < 0.001	2.29 (1.78–2.93), *P* < 0.001	2.32 (1.80–2.99), *P* < 0.001	1.95 (1.54–2.47), *P* < 0.001	2.09 (1.63–2.69), *P* < 0.001
Shelter/displacement	1.00 (0.75–1.35), *P* = 0.98	1.06 (0.78–1.43), *P* = 0.71	1.22 (0.97–1.53), *P* = 0.09	1.24 (0.98–1.58), *P* = 0.08	1.29 (1.04–1.61), *P* = 0.02	1.32 (1.06–1.66), *P* = 0.01	1.28 (1.03–1.59), *P* = 0.03	1.41 (1.12–1.78), *P* = 0.003
Social exclusion	1.21 (0.91–1.61), *P* = 0.19	1.23 (0.92–1.64), *P* = 0.16	1.52 (1.20–1.93), *P* = 0.001	1.57 (1.23–2.02), *P* < 0.001	2.32 (1.81–2.98), *P* < 0.001	2.36 (1.84–3.03), *P* < 0.001	1.68 (1.34–2.10), *P* < 0.001	1.78 (1.40–2.26), *P* < 0.001

CI, confidence interval; OR, odds ratio.

a Covariates included in the model: sex and age.

### Associations with health-related quality of life

Among 331 EQ-5D respondents, the proportion reporting problems in each domain was as follows: 28.4% for mobility, 10.9% for self-care, 29.3% for usual activities, 52.3% for pain/discomfort and 46.8% for anxiety/depression (Supplementary Table 6). The median (IQR) score for EQ-5D health index was 0.92 (0.78–1.00).

Higher ‘livelihood’ and ‘social exclusion’ *z* scores were associated with increased odds of experiencing problems in all five domains, particularly in the usual activities [‘livelihood’: aOR 2.26 (95% CI 1.74–2.93) and ‘social exclusion’: aOR 1.68 (1.32–2.14)] and anxiety/depression [‘livelihood’: aOR 1.94 (1.52–2.47) and ‘social exclusion’: aOR 2.11 (1.66–2.70)] domains (Table 4). In contrast, higher ‘shelter/displacement’ *z* scores were only significantly associated with problems in the usual activities [aOR 1.30 (1.04–1.64)] domain. All three patterns were associated with lower EQ-5D health index scores [‘livelihood’: aBeta −0.11 (−0.13 to −0.08), ‘shelter/displacement’: aBeta −0.04 (−0.06 to −0.01) and ‘social exclusion’: aBeta −0.07 (−0.10 to −0.05)].

**Table 4:** Associations between social determinants of health patterns and health-related quality of life, measured using the EQ-5D questionnaire (*n* = 331), assessed using logistic (EQ-5D domains) or linear (EQ-5D index score) regression models.

	EQ-5D domain: mobility		EQ-5D domain: self-care		EQ-5D domain: unusual activities	
Social determinants of health patterns OR/beta^*^ estimate (95% CI), *P* value	Unadjusted	Adjusted^a^	Unadjusted	Adjusted^a^	Unadjusted	Adjusted^a^
Livelihood	1.78 (1.40–2.27), *P* < 0.001	1.95 (1.50–2.54), *P* < 0.001	2.06 (1.48–2.86), *P* < 0.001	2.20 (1.55–3.13), *P* < 0.001	2.21 (1.72–2.84), *P* < 0.001	2.26 (1.74–2.93), *P* < 0.001
Shelter/displacement	1.06 (0.84–1.34), *P* = 0.63	1.17 (0.91–1.50), *P* = 0.23	1.15 (0.85–1.58), *P* = 0.37	1.29 (0.93–1.80), *P* = 0.13	1.27 (1.01–1.58), *P* = 0.04	1.30 (1.04–1.64), *P* = 0.02
Social exclusion	1.41 (1.12–1.79), *P* = 0.004	1.52 (1.18–1.96), *P* < 0.001	1.42 (1.02–1.97), *P* = 0.04	1.50 (1.06–2.12), *P* = 0.02	1.64 (1.29–2.07), *P* < 0.001	1.68 (1.32–2.14), *P* < 0.001

Social determinants of health patterns	EQ-5D domain: pain/discomfort		EQ-5D domain: anxiety/depression		EQ-5D index score^*^	
	Unadjusted	Adjusted^a^	Unadjusted	Adjusted^a^	Unadjusted	Adjusted^a^
Livelihood	1.55 (1.23–1.94), *P* < 0.001	1.59 (1.25–2.02), *P* < 0.001	1.95 (1.53–2.48), *P* < 0.001	1.94 (1.52–2.47), *P* < 0.001	−0.11 (−0.13 to −0.08), *P* < 0.001	−0.11 (−0.13 to −0.08), *P* < 0.001
Shelter/displacement	1.19 (0.95–1.48), *P* = 0.13	1.24 (0.98–1.57), *P* = 0.07	1.18 (0.95–1.47), *P* = 0.14	1.16 (0.93–1.45), *P* = 0.18	−0.03 (−0.06 to 0.00), *P* = 0.02	−0.04 (−0.06 to −0.01), *P* = 0.01
Social exclusion	1.55 (1.23–1.94), *P* < 0.001	1.62 (1.28–2.05), *P* < 0.001	2.11 (1.66–2.69), *P* < 0.001	2.11 (1.66–2.70), *P* < 0.001	−0.08 (−0.10 to −0.05), *P* < 0.001	−0.07 (−0.10 to −0.05), *P* < 0.001

* Linear regression model.

a Covariates included in the model: sex and age.

### Summary of qualitative findings

The qualitative data contextualized the quantitative patterns, showing how intersecting SDoH shape health outcomes for individuals with HIV (see Table 5 for exemplar quotes to support findings). Participants described how unstable housing not only affects physical health but also leads to social withdrawal, particularly among those facing racism and poor living conditions. Inadequate housing in deprived areas limited social engagement, worsening mental health and overall well being. Similarly, economic hardship, a key dimension of livelihood shaped by limited employment opportunities and immigration restrictions, created a cycle of poverty, affecting access to nutritious food and essential resources. Participants highlighted the impossible trade-offs between affordability and health, when financial insecurity forces reliance on cheaper, less nutritious food, impacting long-term health outcomes. This economic instability was further reinforced by social exclusion, as stigma around HIV, age, and race isolated individuals from community support and employment. The intersection of these factors, precarious housing, financial hardship and systemic discrimination, demonstrates how multiple SDoH intersect and affected the lived experiences of marginalized individuals, creating barriers to both self-management and broader societal inclusion.

**Table 5:** Selected accounts from focus groups and interview participants linked to main findings.

Shelter/displacement	‘You’re in social housing, probably poor quality social housing in a deprived area which may not facilitate comfortable living for your condition, so all those impact on how well they are able to manage their condition … If your housing has damp and neighbours that are not racially accepting, all those kind of issues means you stay indoors, you don’t interact, so your mental health, your quality of life are all affected by you being in a foreign country… (Interview 1) ‘I think because, you know, first of all is a person is not stable mentally and does not have a place, a permanent place, you know, to live in [accommodation], then [this] does not only affect their condition but affects their whole life really, their whole health, their entire health…(So that's the problem because in that situation a person is living on hand-outs, you know, on the goodwill of other people, so they have no control over their lives… (Interview 3)
Livelihood	‘I can categorically say there is a correlation between having no money and then gaining more weight, because when you have no money you go for the cheapest things that you can find … To have it like a complete meal it would be difficult, so maybe you’d think if I get a one piece chicken and chips would cost me £1.40 and if I’m going to get maybe two pieces of chicken I’ll need to buy maybe vegetables, I need maybe to buy rice, for me to make a complete meal I’ll need £5…. (Focus Group 3) ‘How are you going to be eating healthy if the so-called healthy food is more expensive than you can afford. So that is also a discrimination or a stigma … there is no escape route.’ (Interview 2) ‘Well, the thing is the impact of immigration is ongoing, and it is not only something that you expect will impact a person for one year or two, sometimes can go on for a very long time so, and that stops them from working because some people are qualified, they have skills but because of their immigration status they cannot work. And so, they sink in poverty and also families with children because of their immigration status they will not get the same level of benefit … it becomes a vicious circle… (Interview 3)
Social isolation/exclusion	‘….because with ill health you cannot be employed, there are so many disadvantages. If you’re out of a job, God knows how you are going to provide for yourself, live alone, [and you are] depending on you[rself]. Secondly, also because with these kinds of complex illnesses you will no longer be able to do things you used to be able to’. (Focus group 4) ‘All of us have got experience of discrimination. Number 1) being HIV positive because there are a lot of people who still don’t understand or know what HIV is and how is this transmitted…You become suddenly untouchable because people even then fear because they don’t know whether you can get it from touching a person … Age is secondary to that. The older you are, I think society these days doesn’t impress the welfare of the aged, they are discriminated and actually even considered less valuable…. Then the race itself. There's still people who are intolerant…I think, by and large, people who are HIV, African origin, old and then low income have got all of those working against them’ (Interview 2)

Eleven women and nine men participated in mixed-gender focus group discussion (summary of participant characteristics can be found in Supplementary Table 7 [16]), with same-gender breakout discussions for sensitive topics such as reproductive and urological health, including menopause symptoms. There was strong convergence between the accounts of focus group discussion participants and staff, with few gender differences identified.

## Discussion

We identified three patterns of SDoH among people of Black ethnicities living with HIV: ‘livelihood’ (comprising employment, food, and financial insecurity and social isolation); ‘shelter/displacement’ (comprising housing, migration and food insecurity); and ‘social exclusion’ (comprising discrimination, loneliness and isolation). These findings suggest that SDoH cluster in specific, overlapping patterns rather than occurring randomly, with food insecurity appearing in multiple patterns, highlighting the complex and interconnected nature of social disadvantages in real-world settings rather than mutually exclusive groups. This clustering likely reflects broader structural and historical factors, including socioeconomic inequalities, systemic racism and migration dynamics that disproportionately affect racially marginalized people with HIV [31].

Previous studies have shown associations between individual SDoH and depressive symptoms and anxiety in people with HIV [32,33]. Additionally, income and housing insecurity have been linked to increased levels of HIV stigma, which, in turn, may impact mental health negatively [34–36]. Our findings build on this literature by highlighting the cumulative effects of multiple SDoH on health outcomes. All three SDoH patterns were associated with poor mental health, with the strongest association seen with the ‘social exclusion’ pattern. The ‘livelihood’ and ‘social exclusion’ patterns were also associated with lung disease, chronic pain and multimorbidity, suggesting that overlapping social/economic disadvantages may act synergistically to worsen health outcomes, potentially through psychological stressors, reduced healthcare access or environmental exposures such as air quality [37–39]. Given the cross-sectional design, causality cannot be determined. It is plausible that poor health may worsen socioeconomic conditions, such as employment insecurity and social isolation, creating a bidirectional cycle that compounds disadvantage [40,41]. Both ‘livelihood’ and ‘social exclusion’ patterns were linked to worse outcomes across all HRQoL dimensions, suggesting that economic and social disadvantage appear to be strong drivers of poor QoL. These quantitative findings are strongly echoed in the qualitative narratives, which illustrate how interrelated SDoH, such as financial hardship, discrimination and housing instability, compound health challenges and shape HRQoL. Together, our findings emphasize the need for integrated interventions addressing multiple SDoH simultaneously, such as programmes combining financial, housing and mental health support. Strengthening social networks and reducing exclusion may be particularly effective, given their potential to mitigate depressive symptoms and improve overall HRQoL [42].

In contrast, no associations were found between SDoH patterns and CVD, kidney disease or diabetes, which is consistent with earlier work [22]. This suggests that combinations of SDoH may exert differential influence on physical vs. mental-health-related comorbidities. This may also reflect routine HIV care in the UK, that is, regular access to monitoring and preventive care, which could mitigate the impact of SDoH on physical health. Factors such as ART adherence, lifestyle factors (e.g. smoking and alcohol use), and HIV-related inflammation may play a greater role in the development of these comorbidities. It is also possible that we lacked sufficient statistical power to detect associations, warranting further investigation in larger cohorts. No strong associations were observed between low education status and the other SDoHs. This may reflect the small number of participants with lower educational attainment, which could be a result of selective migration processes. Additionally, regular engagement with HIV care may have supported health literacy and access to resources, potentially mitigating the impact of lower education levels on other SDoH factors.

The underlying mechanisms driving associations between SDoH patterns and health outcomes are likely to be complex. One potential pathway is accelerated biological ageing due to persistent exposure to socioeconomic adversity through mechanisms involving long-term inflammation, immune dysregulation, and heightened allostatic load, a process known as ‘weathering’ [43–45]. Longitudinal studies incorporating biomarkers and predictive modelling could clarify these pathways and identify high-risk individuals for targeted intervention.

### Strengths and limitations

This study has several key strengths. Firstly, the use of PCA to identify distinct SDoH patterns and understand how different social determinants cluster and influence health outcomes. Analyses often focus on individual SDoH and their associations with clinical outcomes, rather than reflecting the reality of people's lives where multiple SDoH co-exist and shape health and wellbeing. Secondly, we examined a wide range of SDoH within a well characterized cohort of individuals of Black ethnicities living with HIV in South London, a region that is one of the most ethnically diverse and socioeconomically deprived areas in the UK [46]. Thirdly, our study includes mental health and chronic pain in our definition of multimorbidity, which are often overlooked as comorbidities in existing studies among people with HIV. Fourthly, this study includes measures of HIV-related stigma and generalized discrimination as SDoH, factors particularly relevant to this population, as well as the use of validated tools to measure QoL in this population. Lastly, our mixed-methods design harnesses the strengths of quantitative and qualitative approaches. Whilst the quantitative results highlight how SDoH intersect and are associated with multimorbidity and lower QoL in people of Black ethnicities living with HIV, the qualitative findings capture the nuanced, intersectional nature of these experiences through participants’ lived experiences.

There are some limitations that need to be considered. First, the cross-sectional nature of our study limits our ability to determine causal relationships between SDoH patterns and health outcomes. Second, our cohort consisted exclusively of people of Black ethnicities in the UK with longstanding, well controlled HIV infection, recruited from the GEN-AFRICA study. Therefore, participants with inconsistent or poor engagement in HIV care may have been underrepresented, and our findings may not be generalizable to other HIV populations in the UK. Additionally, the qualitative study findings focus on people of Black ethnicities aged, aged less than 35 years and living outside London, and may, therefore, not be generalizable to other populations living with HIV. Third, data collection was conducted during the COVID-19 pandemic, which may have influenced the SDoH status of some participants. Fourth, medical history was self-reported and could not be validated against medical records, which introduces the potential for recall and misclassification bias, possibly leading to under- or overestimation of comorbidities. Fifth, our ability to adjust for additional confounders (beyond age and sex) was limited by the small sample size, missing data and low variability in relevant covariates, highlighting the need for larger studies to validate these findings and enable more robust adjustment for confounding. Finally, our study did not explore potential mediation effects, which could provide valuable insights into the mechanisms linking SDoH patterns to health outcomes. This may include the mediating role of long-term stress, stigma of HIV and social support in the associations between SDoH patterns and HRQoL. Future research could consider mediation analyses to better understand these pathways.

In conclusion, our findings emphasize the role of intersectionality-informed quantitative and qualitative analyses of SDoH. We describe the multidimensional nature of socioeconomic marginalization among people of Black ethnicities living with HIV in South London, highlighting the substantial burden of socioeconomic deprivation and its role in shaping a range of health outcomes in this population. Interventions targeted at upstream factors need to be multilevel and address multiple SDoH concurrently if they are to maximize impact on the health and well being of marginalized communities living with HIV [47].

## Acknowledgements

The authors would like to thank the study participants, members of the CKD-AFRICA study group [Guy's and St Thomas’ Hospital, London: Julie Fox (PI), Anele Waters; King's College Hospital, London: Frank Post (CI), Rachel Hung, Laura Cechin, Vlad Kolodin, Beatriz Santana-Suarez, Leigh McQueen, Lucy Campbell, Bee Barbini, Emily Wandolo, Kate Childs, Sarah Barber; St. Georges University Hospitals, London: Lisa Hamzah (PI), Katie Toler; Africa Advocacy Foundation: Denis Onyango]; and NIHR HPRU Steering Committee: Professor C.A. Sabin (HPRU Director), Dr J. Saunders (UKHSA Lead), Professor C. Mercer, Dr H. Mohammed, Professor G. Rait, Dr R. Simmons, Professor W. Rosenberg, Dr T. Mbisa, Professor R. Raine, Dr S. Mandal, Dr R. Yu, Dr S. Ijaz, Dr F. Lorencatto, Dr R. Hunter, Dr K. Foster and Dr M. Tahir.

Ethics approval: the quantitative study was approved by the National Health Service Research Ethics Committee (20/LO/0946) and the Health Research Authority (Integrated Research Application System (IRAS) 278244). The qualitative component was approved by the Kings College London Research Ethics Committee (Reference HR/DP-21/22-26315).

Patient consent: all participants provided written informed consent.

Consent for publication: not applicable.

Funding statement: the GEN-AFRICA study was supported by the Medical Research Council (UK) Confidence in Concept scheme (MC_PC_17164), and the CKD-AFRICA study by the Guy's and St Thomas’ Charity Multiple Long Term Conditions Challenge Fund (EIC180702). L.S. was funded through the National Institute for Health and Care Research Health Protection Research Unit (NIHR HPRU) in Blood Borne and Sexually Transmitted Infections at University College London in partnership with the UK Health Security Agency (HPRU Grant no: NIHR200911). The funders had no input in the design or conduct of the study, and were not involved in writing of the manuscript or the decision to publish our findings.

### Conflicts of interest

F.A.P. reports grants and/or personal fees from Gilead Sciences, ViiV Healthcare and MSD; all outside of the work reported here. C.S. has received funding from Gilead Sciences, ViiV Healthcare, MSD and Janssen-Cilag for membership of Advisory Boards and for preparation of educational materials. E.N. has received speaker honoraria/participated in advisory boards for UCB, Pfizer, Gilead, Galapagos, AbbVie, Eli Lilly, Alfasigma, Fresenius, Novartis and has held research grants from Pfizer and Eli Lilly. The funders were not involved in the study design, collection, analysis, interpretation of data, the writing of this article or the decision to submit it for publication. All authors declare no other competing interests.

## Supplementary Material

Supplemental Digital Content

## Data Availability

The database contains personal and sensitive information and is, therefore, not publicly available. Access to the study data and/or samples is governed by the National Health Service data access policy and those of King's College Hospital NHS Foundation Trust, the study sponsor. The Gen-AFRICA and CKD-AFRICA studies are open to collaborations, and all requests from researchers who meet the criteria for access to fully anonymized patient level data will be considered. Concepts can be submitted for review to the principal investigator (Professor Frank Post; E-Mail: frank.post@kcl.ac.uk).
