# Supplementary material for: Intersecting social determinants of health, multimorbidity and quality of life in people of Black ethnicities with HIV in South London
Source: AIDS. 2025 Sep 17;39(15):2305–16. doi: 10.1097/QAD.0000000000004343 (PMC12629123; doi:10.1097/QAD.0000000000004343)
Supplement: Supplemental Digital Content [file aids-39-2305-s001.docx]

**Intersecting social determinants of health, multimorbidity and quality of life in people of Black ethnicities with HIV in South London: a mixed-methods study**

**SUPPLEMENTARY MATERIAL**

**Supplementary Table 1.** Definitions of the social determinants of health

| **Variable** | **Type** | **Definition** |
| --- | --- | --- |
| Financial insecurity | Composite | - Being behind with some or most bills, or - Not having enough money to meet basic needs or having enough money to meet basic needs only some of the time, or - Having needed financial support in the last year |
| Food insecurity | Simple | Having needed food support in the last year |
| Housing insecurity | Simple | Living in a temporary accommodation, living with friends or family, or being homeless |
| Migration status insecurity | Composite | - Having a temporary or limited leave to remain or having no legal status in the UK, or - Having needed immigration support in the last year |
| Job insecurity | Composite | - Being unemployed, sick, or disabled, or - Having needed employment support in the last year |
| Low educational status | Simple | O levels (age 16) or less |
| Social isolation | Composite | - Being alone or isolated, defined as feeling often:   - a lack of companionship, or  - left out or isolated from others, or  - lonely, or   - Having no one to turn to for emotional support, or - Having needed support with loneliness/isolation in the last year |
| Discrimination | Composite | - Feeling little or not at all fairly treated (as per the DISCUSS questionnaire)   - by friends, in dating situations, in starting a family or in their social life in general, or  - with regards to housing, education or job opportunities, or  - with regards to respect to their privacy or personal security and safety, or   - Feeling avoided because of the HIV status |

**Supplementary Table 2.** Definitions of co-morbidities and multimorbidity

| **Co-morbidity** | **Type** | **Definition** |
| --- | --- | --- |
| Systemic hypertension | Composite | - Mean of the second and third readings of SBP ≥140 mmHg and/or - Mean of the second and third readings of DBP ≥90 mmHg and/or - Being on antihypertensive medication |
| Diabetes mellitus | Composite | - HbA1c 6.5% and/or - Being on hypoglycaemic medication |
| Kidney disease | Composite | - eGFR < 60 ml/min/1.73 m^2^ and/or - albumin/creatinine ratio >3 mg/mmol |
| Cardiovascular disease | Composite | Arterial disease, defined as   - Diagnosis of cardiovascular or cerebrovascular accident according to medical history or the stroke questionnaire; or - Diagnosis of congestive cardiac failure, or   Venous disease, defined as   - Having been diagnosed with deep venous thrombosis or pulmonary embolism |
| Lung disease | Composite | - Having a diagnosis of asthma, chronic obstructive pulmonary disease, or interstitial lung disease; or - Having symptomatic lung disease, i.e.: cough, phlegm, shortness of breath and/or wheezing on several or most days |
| Chronic pain | Composite | - Pain present for >1 year, or - Attending pain clinic, or - Having moderate, severe, extreme pain resulting in time off work, seeing GP, or requiring analgesia |
| Poor mental health | Composite | - HADS score for anxiety and/or depression >11; or - Self-reported moderate, severe, or extreme anxiety and/or depression; or - On medication for anxiety, depression, or other psychiatric illnesses |
| Multimorbidity | Simple | >2 of diabetes mellitus, kidney disease, cardiovascular disease, lung disease, poor mental health, and chronic pain (each counted as separate co-morbidities) |

**Supplementary Table 3.** Comparison of baseline socio-demographic, lifestyle and HIV-related characteristics between the final analytic sample (n=340) and those excluded from the overall AFRICA-CKD study sample (n=398)

| **Characteristic** n (%) or median (IQR) | **Final sample** (n=340) | **Excluded sample** (n=58) |
| --- | --- | --- |
| Age (years) | 52 (45 - 57) | 52 (44 - 57) |
| Gender |  |  |
| Male | 158 (46.5) | 22 (37.9) |
| Female | 182 (53.5) | 36 (62.1) |
| Region of birth |  |  |
| Sub-Saharan Africa | 247 (72.7) | 41 (70.7) |
| Caribbean | 32 (9.4) | 5 (8.6) |
| UK/Other | 61 (17.9) | 12 (20.7) |
| BMI (kg/m^2^) | 29.9 (26.6 - 34.3) | 30.7 (25.3 - 34.2) |
| Current smoker | 30 (9.2) | 6 (10.9) |
| HIV-related factors |  |  |
| Years since HIV diagnosis | 14 (9 – 18) | 13 (10-17) |
| On ART | 339 (99.7) | 56 (96.6) |
| Recent CD4+ T-cell count (cells/mm^3^) | 544 (368 - 749) | 561 (394 - 751) |
| Nadir CD4+ cell count | 151 (62 – 275) | 192 (99 - 281) |
| HIV RNA (<200 copies/ml) | 324 (95.3) | 51 (87.9) |

**Supplementary Table 4.** Pairwise associations between the eight social determinants of health, measured using the Somers’ D statistic.

|  | **Financial insecurity** | **Food insecurity** | **Housing insecurity** | **Migration insecurity** | **Employment insecurity** | **Low education status** | **Social isolation** |
| --- | --- | --- | --- | --- | --- | --- | --- |
| **Financial insecurity** | 0.000 | 0.355 | 0.140 | 0.326 | 0.311 | 0.180 | 0.268 |
| **Food insecurity** | 0.355 | 0.000 | 0.238 | 0.292 | 0.377 | 0.124 | 0.151 |
| **Housing insecurity** | 0.140 | 0.238 | 0.000 | 0.256 | 0.141 | 0.114 | 0.110 |
| **Migration insecurity** | 0.326 | 0.292 | 0.256 | 0.000 | 0.225 | 0.122 | 0.188 |
| **Employment insecurity** | 0.311 | 0.377 | 0.141 | 0.225 | 0.000 | 0.286 | 0.148 |
| **Low education status** | 0.180 | 0.124 | 0.114 | 0.122 | 0.286 | 0.000 | 0.212 |
| **Social isolation** | 0.268 | 0.151 | 0.110 | 0.198 | 0.148 | 0.212 | 0.000 |

**Supplementary Table 5.** Correlation between social determinants of health patterns’ burden scores, measured using Spearman’s correlation

|  | **Shelter/ displacement** | **Social exclusion** |
| --- | --- | --- |
| **Livelihood** | 0.50 (p<0.001) | 0.41 (p<0.001) |
| **Shelter/displacement** |  | 0.24 (p<0.001) |

**Supplementary Table 6.** Proportion of AFRICA-CKD participants reporting any problems in each of the five EQ-5D domains, and the median (IQR) score for EQ-5D health index

| **Characteristic** n (%) or median (IQR) | **Total** (n=331) |
| --- | --- |
| Any problems on EQ-5D dimensions |  |
| Mobility | 94 (28.4) |
| Self-care | 36 (10.9) |
| Usual activities | 97 (29.3) |
| Pain/discomfort | 173 (52.3) |
| Anxiety/depression | 155 (46.8) |
| EQ-5D health index score | 0.92 (0.78 – 1.00) |

**Supplementary Table 7.** Sociodemographic characteristics of focus group attendees

|  | Women  (n=11) | Men  (n=9) | Total |
| --- | --- | --- | --- |
| Age |  |  |  |
| 36-45 | 4 | 1 | 5 |
| 46-55 | 3 | 2 | 5 |
| 56-65 | 4 | 6 | 10 |
| Regions of birth |  |  |  |
| Southern Africa | 3 | 2 | 5 |
| Eastern Africa | 5 | 6 | 11 |
| Central Africa | 2 | 0 | 2 |
| Western Africa | 0 | 1 | 1 |
| The Caribbean | 1 | 0 | 1 |
| Years living with HIV |  |  |  |
| <10 | 1 | 0 | 1 |
| 11-20 | 3 | 1 | 4 |
| >21 | 5 | 7 | 12 |
| Not answered | 2 | 1 | 3 |
| Long-term conditions (self-reported)^a^ |  |  |  |
| HIV+ 1 long-term condition | 4 | 2 | 6 |
| HIV+ >1 long-term condition | 5 | 3 | 8 |
| Cardiovascular disease | 6 | 3 | 9 |
| Liver disease | 1 | 0 | 1 |
| Mental illness | 2 | 0 | 2 |
| Diabetes | 2 | 1 | 3 |
| Neuropathy | 1 | 1 | 2 |
| NA (no long-term conditions) | 2 | 4 | 4 |
| Years living with long-term conditions^a^ |  |  |  |
| <5 | 1 | 0 | 1 |
| 6-20 | 3 | 1 | 4 |
| >21 | 6 | 5 | 11 |
| NA | 0 | 1 | 1 |
| Highest level of completed education^a^ |  |  |  |
| No formal | 0 | 1 | 1 |
| Primary | 1 | 0 | 1 |
| Secondary | 4 | 0 | 4 |
| University | 4 | 6 | 10 |
| Employment^a^ |  |  |  |
| Full time | 0 | 0 | 0 |
| Unemployed | 6 | 3 | 9 |
| Part time | 2 | 0 | 2 |
| Voluntary | 1 | 4 | 5 |
| Shared HIV status with others^a^ |  |  |  |
| Family and/or friends only | 1 | 0 | 1 |
| GP, and family and/or friends | 7 | 4 | 11 |
| GP only | 1 | 2 | 3 |
| NA^a^ (not told anyone) |  | 1 | 1 |
| Abbreviations: GP, general practitioner; HIV, human immunodeficiency virus, NA, no LTC, apart from HIV  ^a^ Missing data, participants did not complete all the sociodemographic information | | | |
